# The use of lithic raw materials at the Early Mesolithic open‐air site Feuersteinacker (Vogelsbergkreis, Germany)

**DOI:** 10.1002/gea.21828

**Published:** 2020-10-23

**Authors:** Thomas Hess, Felix Riede

**Affiliations:** ^1^ Department of Archaeology and Heritage Studies University of Aarhus Højbjerg Denmark

**Keywords:** early Mesolithic, lithic raw materials, mobility, petrography, provenance analysis, subsistence

## Abstract

The open‐air site Feuersteinacker near Stumpertenrod has yielded one of the largest lithic assemblages in Central Germany. It repeatedly served as a workshop for the production of stone tools during an early phase of the Mesolithic. The range of lithic raw materials is extremely diverse, but until today, there is only a limited number of archaeological studies on the occurrence of lithic resources in the area. The following study presents the first in‐depth investigation of the use of different rock types by Mesolithic hunter–fisher–gatherers at the site. Provenance analyses using petrographic methods permit raw materials to be assigned to a specific source and provide new insights into their formation. Furthermore, this study explores the way in which the materials were processed throughout the reduction sequence. A comparison of topographic parameters suggests that the location was situated on an important transit route during prehistoric times. The presented results contribute to a better understanding of mobility patterns and subsistence strategies of Early Mesolithic groups in Central Germany.

## 1 INTRODUCTION

### 1.1 Topography

**Figure 1:**
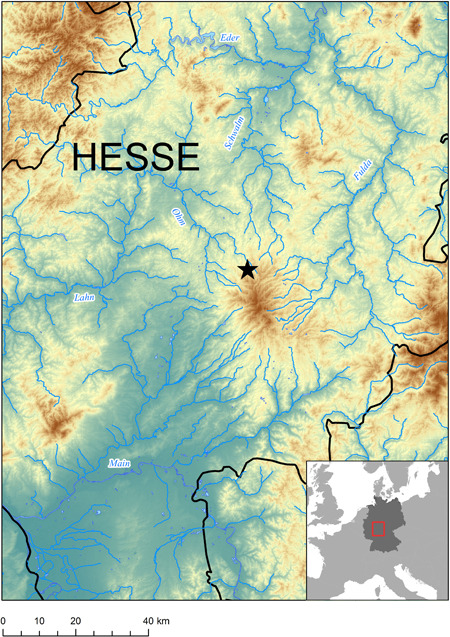
Map showing the location of the open‐air site Feuersteinacker [Color figure can be viewed at wileyonlinelibrary.com]

**Figure 2:**
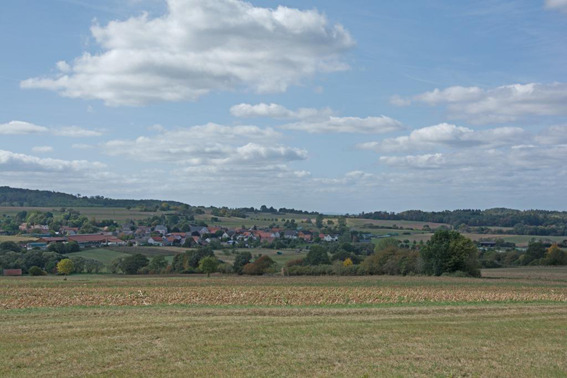
View from the site to the southwest [Color figure can be viewed at wileyonlinelibrary.com]

**Figure 3:**
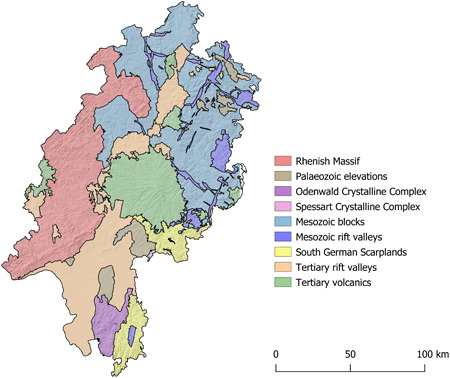
Geological map of the study area [Color figure can be viewed at wileyonlinelibrary.com]

The Mesolithic site Feuersteinacker (Stumpertenrod, Vogelsbergkreis) is located in the central part of the Federal State of Hesse, about 65 km northeast of Frankfurt am Main as the crow flies (Figure 1). It is situated on a gentle southwest‐facing slope near the small village of Stumpertenrod at an elevation of 440 m above sea level. The soil is composed of clay and silt in combination with fine to medium gravel. On both sides of the alluvial terrace, which is now used for agriculture, there are small streams that supply the area with freshwater. Further to the south and the southwest, there is an elongated hill range with a wind power station on top (Figure 2). The Vogelsberg is the largest volcanic structure in Central Europe, and the bedrock in the wider region consists of several superimposed basalt formations (Lotz, 1995, p. 125; Figure 3). Several rivers—flowing radially in all directions—rise in this mountain range. Due to its topographic features, the site is located on a strategically important junction. At the same time, it is protected from storms and floods. Finally, the slightly elevated position presumably allowed prehistoric hunter‐gatherers to observe and approach their prey without being detected (Krüger & Taute, 1964, p. 21).

### 1.2 Research history

The uncannily apt name «Feuersteinacker» («flint field» or «flint acre») derives from the fact that the inhabitants of the nearby village collected siliceous rocks in the field to use as strike‐a‐lights in historical times (Krüger & Taute, 1964, p. 19). After the first antiquarian, collectors had become interested in the site, schoolchildren sold the lithic finds to earn pocket money (personal communication by M. Semmler, September 2019). In 1962, the local farmer Willi Dietz contacted Wolfgang Taute—one of the most important figures in Mesolithic archaeology of the past century—and handed over a surface collection of nearly 400 finds to the museum in Gießen. It soon became clear that the site was of great archaeological relevance. Therefore, the location was inspected on a regular basis each year. Within an area of almost 1 ha, thousands of surface finds were collected. Between 1964 and 1966, a team under the auspices of Wolfgang Taute conducted a test excavation. The investigated area included a 32‐m‐long trench with a width of 1 m (Krüger & Taute, 1964, pp. 24–27). The entire excavated sediment was screened. As a consequence of ploughing, the archaeological features were not in situ anymore and the excavators did not detect any further structures. However, the site that was typologically dated to the Beuronien A (∼9000 BC) forms an important reference point for Mesolithic research in Germany. According to palynological data and radiocarbon dates from the nearby Lahn Valley, there was an increased human impact during the Preboreal and the early Boreal (Bos & Urz, 2003). The environment was characterised by broad meandering rivers and marshlands in combination with open forests, dominated by hazel on the terraces. As many locations in Hesse yielded mixed assemblages—that are the results of several occupations dating to different time periods—the study contributed significantly to a better understanding of the archaeological record in the region. In addition to a report summarising the results of the field campaign (including a description of the stratigraphic sequence), the site is mentioned in Taute's habilitation treatise which was aimed at creating a chronological scheme for the Mesolithic in Southwestern Germany and adjacent areas (Taute, 1971, pp. 282–284). During the following years, the amateur archaeologist Horst Quehl conducted extensive field surveys with the permission of the responsible authorities. Until 2016, he collected more than 7000 lithic artefacts (Fiedler, 2017). Between 2019 and 2020, the assemblage discovered during the excavation, as well as the surface finds were systematically analysed in the framework of an Early Postdoc.Mobility fellowship funded by the Swiss National Science Foundation.

## 2 MATERIALS AND METHODS

### 2.1 Provenance analysis

Provenance analyses of the lithic raw material are of particular interest for the reconstruction of prehistoric territories and mobility patterns (Affolter & Nielsen, 2006; Eriksen, 2002; Högberg & Olausson, 2007; Kind, 2006; Moreau et al., 2015). Especially in case of sites with poor preservation of organic materials, the study of stone tools is fundamental to address the mentioned issues. Lithic sourcing was conducted using a Zeiss Stemi 2000 optical microscope with a magnification of up to ×80. Each artefact was described petrographically with reflected light and subsequently compared to a reference collection. An important advantage of this method is the fact that the analysis was nondestructive. The reference collection consists of rock samples that were collected in the framework of field surveys in 2019 and were supplemented with specimens provided by the Hessian State Museum in Kassel. Currently, it comprises 23 different raw materials, including primary sources and secondary deposits, such as river gravels. Each outcrop was documented photographically, and the coordinates were measured with a handheld global positioning system device. Geological maps (Rösing, 1976) and previous work on the issue (Pflug, 1993) served as a starting point for investigations in the field. For a more detailed description of the rocks, selected samples were studied in thin section.

**Figure 4:**
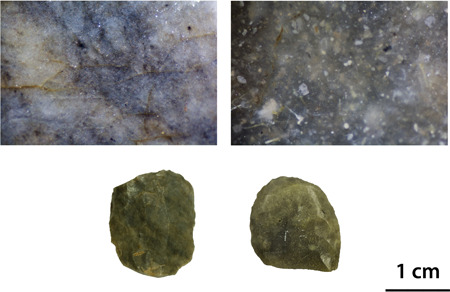
Comparison between the macro‐ and microscopic appearance of two end‐scrapers made of different raw materials (magnification: ×40). Siliceous shale (left) and Cretaceous flint (right) [Color figure can be viewed at wileyonlinelibrary.com]

Several scholars working on the site have pointed out the difficulties in distinguishing the lithic raw materials based on their macroscopic appearance (cf., Fiedler, 2017, pp. 3–4; Taute, 1971, p. 19). Therefore, it was crucial to study the artefacts under a microscope (Figure 4). In the case of biogenic sedimentary rocks, the microfacies analysis was applied (Flügel, 1982, 2010). Originally developed for the study of carbonate rocks, it involves a detailed description of the texture and the components of a sample. The occurrence of microfossils and their degree of preservation allow a reconstruction of the depositional setting in which the rock was formed. Thus, it is possible to distinguish between a nonmarine environment, such as a freshwater lake, and different marine zones, for e.g. a coastal milieu, a reef, a continental slope, or a pelagic milieu. As flint and chert occur within carbonate rock formations and the original texture of the parent material is preserved when it is replaced by silica, the criteria for their petrographic description are mostly the same. The mentioned raw material samples were classified according to the work of Dunham (1962). Archaeological applications of microfacies analysis have already produced interesting results concerning the procurement and circulation of lithic raw materials in Switzerland and Southwestern Germany (Affolter, 2002; Hess, 2019; Kaiser, 2013).

The petrographic description of silicified sandstone *(Tertiärquarzit)* was based on classification schemes developed for the study of clastic sedimentary rocks (Füchtbauer, 1974; Tucker, 1992). In this case, it was possible to distinguish different raw materials based on parameters like grain size, sorting, and roundness. As these aspects are considered to be a function of the transport distance of components, they also contain spatial information.

In addition to the texture of lithic materials, the properties of the cortex were considered. It was possible to distinguish between a primary cortex (i.e., fresh or chalky), a battered surface—which is typical for transport by rivers or moraines—and a cortex that is the result of chemical weathering (Affolter, 2002, p. 19; Kaiser, 2013, p. 81). The colour of the raw materials was determined using the Munsell Color Chart.

### 2.2 Sample

The archaeological sample considered in the framework of this study consists of surface finds in the collection of the Hessian State Museum in Kassel and objects that are displayed in the permanent exhibition of the museum. It includes 8089 artefacts (≥1 cm). The objects were sorted by raw material and assigned to different artefact classes. For a better understanding of the raw material economy, the number of cortical pieces was determined for each rock type. The weight of the pieces was measured with a precision scale (accuracy: 0.1 g). Although it has been subject to postdepositional processes, the assemblage dates almost exclusively to an early phase of the Mesolithic, corresponding to the Beuronian A in Southwestern Germany (Taute, 1971, p. 282). Artefacts with typical features of other time periods were not considered in this study. With a total of 2076 pieces, the largest part of the assemblage consists of preparation artefacts. These are followed by shatter debitage, with 432 objects. In addition, there are 367 modification products (such as microburins) in the assemblage. The blanks consist of 3167 flakes, 330 blades, 591 bladelets, and 241 pieces that could not be further determined. Moreover, it was possible to document 339 cores, including unipolar, bipolar, prismatic, and centripetal examples. With a number of 546 artefacts, the percentage of modified pieces is relatively high (Table 1). These consist of microliths, notched pieces, laterally retouched pieces, end‐scrapers, burins, truncations, semifinished products of microliths, perforators, multifunctional tools, and denticulates (Table 2). The microliths include oblique truncations, triangles, backed bladelets, and crescents.

**Table 1:** Overview over the assemblage (*n *= 8089)

Artefact class	Number	Percentage
Cores	339	4.2%
Preparation artefacts	2076	25.7%
Flakes	3167	39.2%
Blades	330	4.1%
Bladelets	591	7.3%
Undetermined blanks	241	3.0%
Shatter debitage	432	5.3%
Modification products	367	4.5%
Modified pieces	546	6.7%
Total	8089	100%

**Table 2:** Overview over the modified pieces within the assemblage (*n* = 546)

Artefact class	Number	Percentage
Microliths	329	60.3%
Notched pieces	62	11.4%
Laterally retouched pieces	46	8.4%
Scrapers	37	6.8%
Burins	23	4.2%
Truncations	17	3.1%
Semifinished products	12	2.2%
Perforators	9	1.6%
Multifunctional tools	8	1.5%
Denticulates	3	0.5%
Total	546	100%

## 3 RESULTS

### 3.1 Provenance analysis

**Figure 5:**
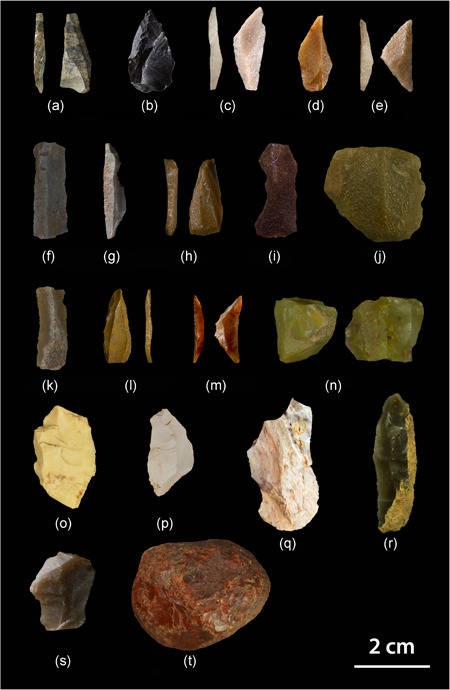
Lithic artefacts found at the Mesolithic open‐air site Feuersteinacker. (a,b) Siliceous shale. (c–g) Silicified sandstone (Lenderscheid). (h–k) Silicified sandstone (Rörshain). (j,k) Silicified sandstone, green. (l–n) Chalcedony. (o,p) Jurassic chert. (q) Triassic chert. (r,s) Cretaceous flint. (t) Jasper [Color figure can be viewed at wileyonlinelibrary.com]

**Figure 6:**
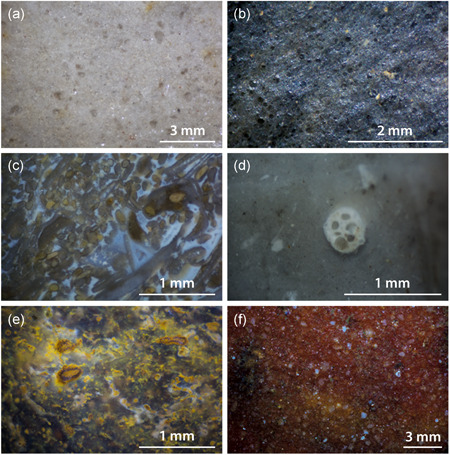
Microscopic images of lithic raw materials present at the site. (a) Silicified sandstone (Lenderscheid). (b) Siliceous shale. (c) Triassic chert. (d) Cretaceous flint. (e) Chalcedony. (f) Silicified sandstone (Rörshain) [Color figure can be viewed at wileyonlinelibrary.com]

The lithic raw material from Feuersteinacker is extraordinarily diverse in terms of its composition. Besides different types of silicified sandstone, chert, and siliceous shales, there is so‐called chalcedony. The spectrum of colours ranges from black through red to white and grey. There are even green materials within the assemblage. In the following, the present rock types will be discussed in more detail (Figures 5 and 6).

#### 3.1.1 Silicified sandstone

Silicified sandstone *(Tertiärquarzit)* was formed under semiarid conditions during the a, by the silicification of sand layers. The silica derives from Tertiary volcanic rocks and was dissolved by groundwater (Freyberg, 1926). In the study area, the material occurs at several places in the shape of large boulders of up to 1 m, as well as smaller nodules. The coloration of silicified sandstones is mainly dependent on the composition of the original sediment. Iron oxide leads to a red colour, while green pieces contain the mineral olivine or epidote. An important outcrop is situated near Lenderscheid, where a particularly fine‐grained and well‐sorted variant is found. Typical colours are white (10.0Y 9/2), grey (5.0Y 5/2), orange (5.0Y 7/20, 2.5YR 6/18), and pink (5.0R 6/12, 5.0R 7/8) (Figures 5c–g and 6a). Other sources are located in Hausen, Ziegenhain, and Rörshain.

#### 3.1.2 Siliceous shale

Local siliceous shale *(Kieselschiefer)* is the most common lithic raw material in the study area. The term refers to a range of different rocks that naturally occur in gravels of major rivers and streams. Primary outcrops can be found in the Rhenish Massif (Figure 3) and the Thuringian Highlands. Siliceous shales are formed in a pelagic environment below the carbonate compensation depth (Füchtbauer, 1974). Petrographically, they can be described as Palaeozoic radiolarian cherts. As a consequence of tectonic stress, there are differences concerning the preservation of microfossils. The colour of the material is a product of carbonaceous pigments (Carozzi, 1960) and ranges from black to dark olive green (5.0Y 2/2, 5.0Y 6/4, 5.0GY 3/4; Figures 5a,b and 6b).

#### 3.1.3 Chalcedony

**Figure 7:**
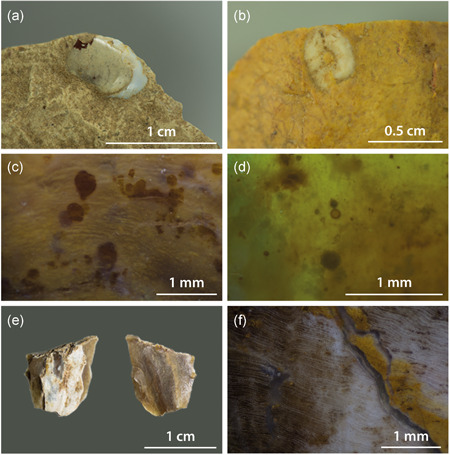
Fossils and microscopic features found in chalcedony. (a,b) Gastropods. (c,d) Algal remains. (e,f) Remains of silicified wood [Color figure can be viewed at wileyonlinelibrary.com]

Another fascinating raw material that is abundant in the study area is chalcedony. It occurs at several locations in Hesse and is associated with basalt formations dating to the Tertiary. Primary outcrops are situated at Steinheim near Frankfurt (Behn, 1925; Deecke, 1933, p. 5), as well as Homberg near the river Ohm. Chalcedony is usually translucent and has a characteristic lustre that is comparable to flint. The spectrum of colours includes light grey (5.0Y 9/2), brown (2.5Y 5/2, 7.5YR 3/2), red (10.0R 3/10, 10.0R 3/8), orange (5.0YR 7/20, 10.0R 6/16), yellow (5.0YR 8/14, 7.5YR 6/12), green (7.5Y 5/6), and light blue (10.0BG 9/2) (Figures 5l–n and 6e). Microscopic analysis revealed that in combination with completely homogeneous parts, characterised by a lack of components, there are microfossils pointing to a lacustrine setting. The latter include gastropods—which are sometimes also part of the cortex—as well as the remains of green algae *(Charophyta)* (cf., Flügel, 2010, pp. 448–450). Furthermore, typical structures of petrified wood were documented. This leads to the conclusion that the material was formed in a Miocene lake by the silicification of submerged trees and algal remains (Figure 7). The silica was dissolved by the chemical weathering of volcanics.

At the same time, it was possible to demonstrate that a raw material, which is commonly referred to as *“Basalthornstein”* in the German‐speaking literature (Fiedler, 2017; Krüger & Taute, 1964; Pflug, 1993), belongs in fact to the same group. As the volcanic rocks in Hesse are the product of multiple eruptive events, chalcedony sometimes occurs as alternating layers within basalt quarries.

#### 3.1.4 Cretaceous flint

The Cretaceous flint recovered at Feuersteinacker is without exception of nonlocal origin. It occurs in moraine deposits of the Saale and Elster glaciation north of 51° N latitude (Floss, 1994, p. 103). The nearest occurrence of Cretaceous flint is in Lower Saxony, where the raw material played an important role during the Mesolithic (Grote, 1993). Other potential sources that were evidentially used by Mesolithic hunter–gatherers are alluvial terraces of the rivers Rhine and Maas (Floss, 1994, p. 98; Gelhausen et al., 2003). Due to their characteristic shape, the pebbles are also called *Maaseier*. The latter often display concentric zoning (so‐called Liesegang rings). They can be classified as wackestones, including triaxon sponge spicules and planktonic foraminifera. Cretaceous flint is usually strongly silicified and translucent. Colours include dark reddish brown (5.0YR 3/4), grey (2.5Y 8/2), and yellow (10.0YR 8/11). Some pieces show a white patination which is the result of fire.

#### 3.1.5 Jurassic chert

In the study area, Jurassic chert occurs in alluvial terraces of the river Main. The nearest primary sources of the material are limestone formations of the Swabian and the Franconian Jura. Due to the transport by alluvial processes, the nodules generally show a battered cortex. The rocks can be described as mud‐ and wackestones pointing to a neritic environment. Microfossils include remains of sponges and corals, bivalves, gastropods, foraminifera, ostracods, bryozoans, as well as crinoids. Typical colours are grey (10.0YR 7/2) and white (5.0Y 9/2). As the geological units and raw material outcrops along the Jura mountain range have been studied in detail, it is relatively easy to identify the respective sources (Affolter, 2002; Altorfer & Affolter, 2011; Binsteiner, 2005; Burkert, 2013; Geyer & Gwinner, 2011; Hess, 2019).

#### 3.1.6 Triassic (Muschelkalk) chert

Local outcrops of Triassic chert are situated in Muschelkalk formations near Tann, to the east of Stumpertenrod (Pflug, 1993, pp. 71–72). Typical components visible under the microscope are ooids and oncoids, suggesting an intertidal environment. In addition, there are packstones containing shells of bivalves and brachiopods (Figure 5c). Other sources of Triassic chert are secondary deposits along the rivers Main and Neckar south of the site (Siegeris, 2010; Spies, 2017, pp. 38–41).

#### 3.1.7 Other raw materials from river gravels (radiolarian chert, quartz, and chert from Flysch formations)

Several lithic raw materials with primary sources in alpine regions are present in river gravels of the Rhine to the south of the site. They include radiolarian chert of Mesozoic age, quartz, as well as chert from Flysch formations.

#### 3.1.8 Jasper (Kellerwald region)

Jasper from the Kellerwald region *(Kellerwald Jaspis/Eisenkiesel)* occurs as secondary deposits in gravels of the river Lahn to the north of Stumpertenrod. It is of Palaeozoic (Devonian/Carboniferous) origin and owes its red and green colour to iron oxide and manganese, respectively. The material is often fissured and contains spherical inclusions. Petrographic analysis suggests that the genesis of the rock is linked to hydrothermal vents (Schneiderhöhn, 1941). It seems to have played only a minor role as a raw material for the production of tools during prehistoric times (see also Pflug, 1993, p. 80).

### 3.2 Raw material economy

**Figure 8:**
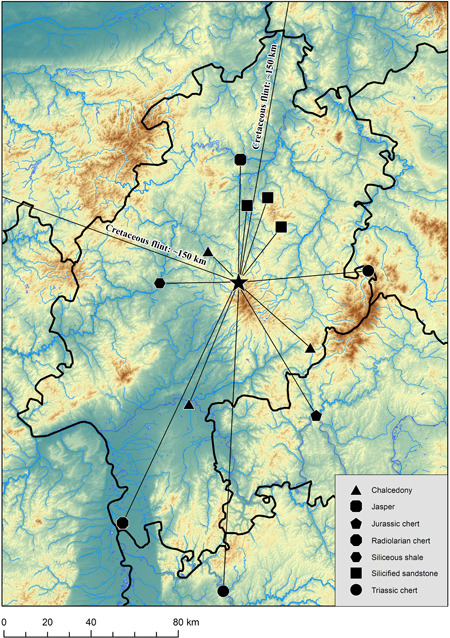
Map showing the origin of lithic raw materials found at the site [Color figure can be viewed at wileyonlinelibrary.com]

**Figure 9:**
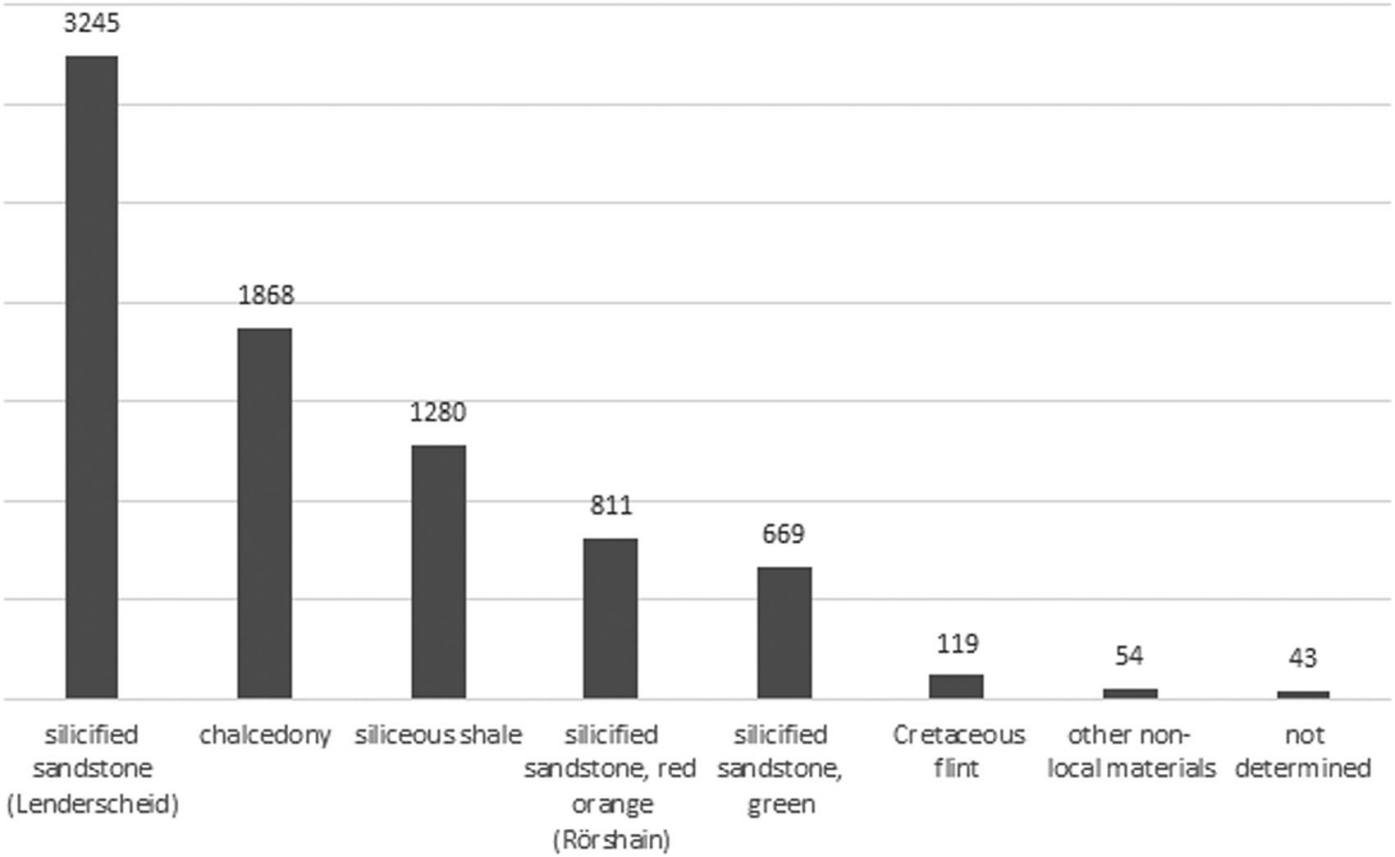
Chart showing the percentage of different raw materials (*n* = 8089)

With a total of 3245 pieces (40.1%), the largest part of the assemblage consists of silicified sandstone from Lenderscheid (Figure 8). The outcrop is situated around 40 km to the northeast of the site, as the crow flies. This is followed by chalcedony with 1868 pieces (23.1%). Potential sources are located in basalt formations in Homberg (Ohm), approximately 20 km northeast of the site and near Steinheim, around 60 km to the southwest. A minor part of the chalcedony within the assemblage could also be assigned to a source near Braunfels an der Lahn. Siliceous shales are represented by 1280 artefacts (15.8%). Material of a suitable size and quality occurs in alluvial terraces of the river Lahn, approximately 30 km to the west. A redtoorange coarse‐grained silicified sandstone that can be found near Rörshain is present with 811 artefacts (10.0%) (Figure 5h,i). A further 669 objects (8.3%) consist of a green variety of silicified sandstone that occurs in the same area (Figure 5j). The primary sources of the mentioned materials are situated at a distance of 50 km from the site. A total of 119 artefacts (1.5%) indicate the use of Cretaceous flint. In this case, it was possible to identify two different sources. Whereas one material points to moraines northeast of the site, there are also pieces that are typical for gravels of the Lower Rhine Bay to the northwest of Stumpertenrod. In both cases, the material was imported from a distance of at least 150 km. A number of 29 objects (0.4%) consist of Jurassic chert. The raw material could either derive from secondary sources near the river Main or from primary outcrops on the Swabian and the Franconian Jura. Unlike Jurassic chert that occurs in the south of Baden‐Württemberg, the artefacts in the assemblage are not secondarily stained by iron ore. In addition, there is a small amount of chert from Muschelkalk formations (seven pieces, 0.1%), 60 km to the east and 130 km to the south of the site. Radiolarian chert, quartz, and chert from Flysch formations (6 pieces, 0.1%) are pointing to river gravels in the south. 6 pieces (0.1%) indicate the use of bluish‐grey silicified sandstone from Ziegenhain in the Schwalm Valley, right next to Rörshain. Furthermore, 3 objects consist of jasper from the Kellerwald region. The material occurs in alluvial terraces of the river Eder, 55 km to the north of Stumpertenrod. Finally, there are 3 pieces of silicified sandstone from Hausen, about 35 km to the northeast of the site. 43 artefacts could not be determined, due to their small size or patination (Figure 9; Table 3).

**Table 3:** Importance of different raw material types at Feuersteinacker by number and weight (*n* = 8089)

Raw material	Number	Percentage	Weight (g)	Percentage
Silicified sandstone (Lenderscheid)	3245	40.1%	5209.5	32.2%
Chalcedony	1868	23.1%	4664.7	28.9%
Siliceous shale	1280	15.8%	1962.1	12.1%
Silicified sandstone, red orange (Rörshain)	811	10.0%	1660.4	10.3%
Silicified sandstone, green	669	8.3%	2106	13.0%
Cretaceous flint	119	1.5%	187.2	1.2%
Jurassic chert	29	0.4%	33.8	0.2%
Triassic chert	7	0.1%	13.1	0.1%
Silicified sandstone, bluish grey (Ziegenhain)	6	0.1%	8.9	0.1%
Materials from river gravels	6	0.1%	31.8	0.2%
Silicified sandstone (Hausen)	3	<0.1%	19.6	0.1%
Jasper (Kellerwald region)	3	<0.1%	52.4	0.3%
Not determined	43	0.5%	206.6	1.3%
Total (*n*)	8089	100%	16,156.1	100%

Tables 4 and 5 show that there are significant differences concerning how the various raw materials were processed. Silicified sandstone from Lenderscheid occurs in the form of large slabs that were intentionally fragmented and transformed into cores. Therefore, the percentage of cortical flakes is smaller than in the case of nodular materials. Cores, blanks, and even microburins made of silicified sandstone from Lenderscheid are relatively large for a Mesolithic assemblage. The former are often polyhedral in shape and show traces of bipolar reduction. Another common core scheme involves nuclei with two adjacent reduction planes. Furthermore, it is important to state that laminar blanks made of silicified sandstone are often more regular, compared to other materials. In the case of siliceous shale, cores frequently show a longitudinal section of trapezoidal shape with angles around 120° and 60°, respectively. This seems to be a direct consequence of the fracture behaviour of the material. The high percentage of preparation flakes indicates that entire nodules of siliceous shale were brought to the site. As chalcedony is often fissured, its use by Mesolithic people at Feuersteinacker displays a higher degree of flexibility. The material was processed in a more economical way, leading to small remaining nuclei (Figure 5n). They have an average weight of 12.1 g (compared to 16.5 g for specimens made of silicified sandstone). Although with 23.1%, chalcedony is only the second‐most common raw material at the site, it accounts for the largest part of cores (46.3%). It is strongly fragmented, and due to its brittleness, there is a higher tendency towards the formation of shatter debitage compared to other rock types. Interestingly, Jurassic and Triassic cherts that were imported from the south show a characteristic alteration of colours, suggesting an intentional heat‐treatment (Figure 5o–q). In combination with typotechnological similarities, this suggests contacts to the Beuronian in Southwestern Germany (cf., Eriksen, 2006). In the case of Jurassic chert, there are no cortical flakes in the assemblage, which leads to the conclusion that the material was transported to the site in the form of prepared cores or artefacts. Cretaceous flint, on the other hand, was brought to the site as entire nodules. A single core of radiolarian chert shows a polyhedral shape. The only pebble consisting of jasper from the Kellerwald region could either have served as a percussion tool or a raw nodule (Figure 5t).

**Table 4:** Contribution of raw material types to different artefact classes

Laminar blanks	Laminar blanks (%)	Shatter debitage	Shatter debitage (%)	Modified pieces	Modified pieces (%)	Microliths	Microliths (%)
363	39.4%	123	28.5%	196	35.9%	151	45.9%
220	23.9%	189	43.8%	164	30.0%	82	24.9%
162	17.6%	41	9.5%	82	15.0%	37	11.2%
70	7.6%	41	9.5%	43	7.9%	32	9.7%
81	8.8%	26	6.0%	31	5.7%	15	4.6%
15	1.6%	2	0.5%	21	3.8%	5	1.5%
4	0.4%	3	0.7%	0	0.0%	0	0.0%
2	0.2%	2	0.5%	0	0.0%	0	0.0%
0	0.0%	0	0.0%	1	0.2%	0	0.0%
0	0.0%	1	0.2%	2	0.4%	2	0.6%
1	0.1%	0	0.0%	1	0.2%	1	0.3%
0	0.0%	0	0.0%	2	0.4%	1	0.3%
3	0.3%	4	0.9%	3	0.5%	3	0.9%
921	100%	432	100%	546	100%	329	100%

**Table 5:** Number of cortical flakes among different raw materials (*n* = 8089)

Raw material	Number	Cortical pieces	Percentage
Silicified sandstone (Lenderscheid)	3245	277	8.5%
Chalcedony	1868	360	19.3%
Siliceous shale	1280	324	25.3%
Silicified sandstone, red orange (Rörshain)	811	164	20.2%
Silicified sandstone, green	669	168	25.1%
Cretaceous flint	119	31	26.1%
Jurassic chert	29	0	0.0%
Triassic chert	7	1	14.3%
Silicified sandstone, bluish grey (Ziegenhain)	6	2	33.3%
Materials from river gravels	6	1	16.7%
Silicified sandstone (Hausen)	3	0	0.0%
Jasper (Kellerwald region)	3	1	33.3%
Not determined	43	16	37.2%

Blades and bladelets made of silicified sandstone from Lenderscheid are slightly underrepresented compared to the overall number of blanks, which could be due to the fact that they were more often transformed into tools. Regarding the 329 microliths in the assemblage, there was a clear preference for materials with a high silica content, such as silicified sandstone from Lenderscheid and chalcedony. These two materials account for more than 70% of this category.

## 4 DISCUSSION

The results presented in this article demonstrate that Mesolithic hunter–fisher–gatherers at Feuersteinacker used a wide variety of lithic raw materials, dominated by silicified sandstone from the Schwalm Valley. They came from all directions and were transported to the site over considerable distances. Whereas the largest part of the assemblage consists of materials that can be found within a range of 60 km, some rock types were imported from distant sources that are up to 150 km away from the site. The spatial distribution of raw material outcrops and other Mesolithic locations in the study area implies that river systems played an important role as traffic routes and axes of communication (in this context see Floss, 2002). Several watercourses originate in the Vogelsberg mountains and what is nowadays a rather remote, rural area seems to have been a major transportation hub during the Mesolithic, and a persistent place in the early Holocene landscape (cf., Barton et al., 1995, p. 81). In this context, it is also interesting to mention archaeobotanical evidence for intentional woodland clearance by fire setting (Bos & Urz, 2003, pp. 31–33).

Large numbers of cores, preparation flakes, microburins, and semifinished products suggest that the site was repeatedly used as a workshop for the serial production of stone tools. Furthermore, several elongated sandstone pebbles that served as retouching tools were discovered. The lithic assemblage analysed in the framework of this study has a weight of more than 16 kg (!). This is a substantial quantity of material, considering that Mesolithic technology was based on the production of microliths. It should also be added that the sample only reflects a small part of the entire lithics discarded at the site. Laminar blanks are more regular compared to contemporary sites in adjacent areas and appear to be standardised. The fact that various raw materials were processed in a different way implies a good understanding of their physical properties and a certain degree of specialisation. Furthermore, the presence of Jurassic chert and Cretaceous flint argue for a far‐reaching contact network. In this context, it is possible to suggest seasonal gatherings of otherwise dispersed groups (cf., Baales, 2001). A technological system involving composite tools offers new possibilities concerning the production and exchange of material culture (Finlay, 2003). As in the case of other Mesolithic sites, it is possible that the colour of siliceous rocks was an additional criterion for their selection. While Jurassic chert in the Early Mesolithic record of Southwestern Germany was intentionally heat treated (Eriksen, 2006; Hess, 2019), lithic raw material that is available in the wider region naturally occurs in a variety of different colours. Together with pigments, rocks were among the most colourful materials in prehistoric times, and ethnographic analogies suggest that in addition to functional aspects, they might have had a symbolic meaning (Hess, 2019; Taçon, 2008).

Finally, there are a number of landscape archaeological implications derived from the observations detailed above. First, and in line with previous studies in neighbouring regions (e.g. Eriksen, 2002; Floss, 2002; Jochim, 1998), the results argue for new patterns of land use emerging with the onset of the Mesolithic in the study area. Transportation routes, linking raw material outcrops and settlements, followed the river systems of Lahn, Main, Fulda, Schwalm, Ohm, and Eder. In addition, mountain ranges (e.g., the Vogelsberg and the Rhön mountains) could have served as important landmarks (cf., Hess, 2019). Mesolithic sites are often situated on elevated terraces that are protected from floods, near small streams leading to the tributary waters of larger rivers (cf., Fiedler, 1994; Pflug, 1993, pp. 35–37; Quehl, 1994). One of them is the site Niederweimar in the Lahn Valley (Schön, 2006; Urz et al., 2002, p. 275). Archaeological excavations yielded microliths as well as pebbles that served as retouching tools comparable to the finds from Feuersteinacker. At this site, the percentage of local raw materials (mainly siliceous shale) is much higher, which indicates shorter stays. The faunal assemblage consists of roe deer, red deer, wild boar, and aurochs (Bos & Urz, 2003, p. 32). Other sites with a similar age that probably served as small hunting camps were discovered at Lahrbach and Kleinsassen (Pflug, 1993, pp. 15–17, 35–37). In both cases, the proportion of local raw materials is higher than 60%. Whereas the assemblage found at Lahrbach consist mainly of Triassic chert from Muschelkalk formations, most lithic artefacts from Kleinsassen are made of chalcedony. Second, it can be shown that the technology of Early Mesolithic groups in Central Germany allowed the use of a broader spectrum of lithic resources compared to previous time periods. Large assemblages dating to the Late Palaeolithic are usually dominated by siliceous shale, which contributes between 50% and 90% to the raw material (Fruth, 1994; Hofbauer, 1992; Loew, 2005; Riede, 2012). The observed differences are connected with shifting settlement dynamics and a reduction of residential mobility (cf., Binford, 1980). In case of the Hessian Late Palaeolithic/Early Mesolithic, these are likely to reflect a shift from a rather ephemeral human presence in the region during the final Pleistocene towards a more sustained land use in the early Holocene. As rock types associated with Tertiary volcanism are frequently weathered when exposed to the surface, it is even possible to suggest that a simple form of mining (such as digging shallow pits) was used to obtain the material. On a large scale, the processes described above can be interpreted as an adaptation to changing environmental factors. In Central Germany, the combined impact of the Laacher See volcanic eruption around 13,000 cal BP, the subsequent Younger Dryas cooling, and the equally dramatic warming in the early Holocene likely led to this reorganisation of mobility and raw material procurement (cf., Riede, 2016).

Future research could focus on integrating the results of provenance analysis into an existing geographic information system‐based predictive model (Sauer et al., 2018) to detect new sites dating to the Late Palaeolithic and the Mesolithic in the study area. This could contribute to a better understanding of the Pleistocene–Holocene transition in the region and lead to an archaeological definition of the respective cultures.

## CONFLICT OF INTERESTS

The authors declare that there are no conflict of interests.

## Data Availability

The data that support the findings of this study are available from the corresponding author upon reasonable request.
